# The HLA Class II Allele DRB1*1501 Is Over-Represented in Patients with Idiopathic Pulmonary Fibrosis

**DOI:** 10.1371/journal.pone.0014715

**Published:** 2011-02-23

**Authors:** Jianmin Xue, Bernadette R. Gochuico, Ahmad Samer Alawad, Carol A. Feghali-Bostwick, Imre Noth, Steven D. Nathan, Glenn D. Rosen, Ivan O. Rosas, Sanja Dacic, Iclal Ocak, Carl R. Fuhrman, Karen T. Cuenco, Mary A. Smith, Susan S. Jacobs, Adriana Zeevi, Penelope A. Morel, Joseph M. Pilewski, Vincent G. Valentine, Kevin F. Gibson, Naftali Kaminski, Frank C. Sciurba, Yingze Zhang, Steven R. Duncan

**Affiliations:** 1 Department of Medicine, University of Pittsburgh, Pittsburgh, Pennsylvania, United States of America; 2 Medical Genetics Branch, National Human Genome Research Institute, National Institutes of Health, Bethesda, Maryland, United States of America; 3 Department of Medicine, University of Chicago Medical Center, Chicago, Illinois, United States of America; 4 Advanced Lung Disease Program, Inova Fairfax Hospital, Falls Church, Virginia, United States of America; 5 Department of Medicine, Stanford University Medical Center, Stanford, California, United States of America; 6 Department of Medicine, Brigham and Women's Hospital, Harvard Medical School, Boston, Massachusetts, United States of America; 7 Department of Pathology, University of Pittsburgh, Pittsburgh, Pennsylvania, United States of America; 8 Department of Radiology, University of Pittsburgh, Pittsburgh, Pennsylvania, United States of America; 9 Department of Oral Biology-Dental Medicine and Human Genetics, University of Pittsburgh, Pittsburgh, Pennsylvania, United States of America; 10 Department of Immunology, University of Pittsburgh, Pittsburgh, Pennsylvania, United States of America; 11 Department of Medicine, University of Texas Medical Branch, Galveston, Texas, United States of America; Comprehensive Pneumology Center, Germany

## Abstract

**Background:**

Idiopathic pulmonary fibrosis (IPF) is a progressive and medically refractory lung disease with a grim prognosis. Although the etiology of IPF remains perplexing, abnormal adaptive immune responses are evident in many afflicted patients. We hypothesized that perturbations of human leukocyte antigen (HLA) allele frequencies, which are often seen among patients with immunologic diseases, may also be present in IPF patients.

**Methods/Principal Findings:**

HLA alleles were determined in subpopulations of IPF and normal subjects using molecular typing methods. HLA-DRB1*15 was over-represented in a discovery cohort of 79 Caucasian IPF subjects who had lung transplantations at the University of Pittsburgh (36.7%) compared to normal reference populations. These findings were prospectively replicated in a validation cohort of 196 additional IPF subjects from four other U.S. medical centers that included both ambulatory patients and lung transplantation recipients. High-resolution typing was used to further define specific HLA-DRB1*15 alleles. DRB1*1501 prevalence in IPF subjects was similar among the 143 ambulatory patients and 132 transplant recipients (31.5% and 34.8%, respectively, p = 0.55). The aggregate prevalence of DRB1*1501 in IPF patients was significantly greater than among 285 healthy controls (33.1% vs. 20.0%, respectively, OR 2.0; 95%CI 1.3–2.9, p = 0.0004). IPF patients with DRB1*1501 (n = 91) tended to have decreased diffusing capacities for carbon monoxide (DL_CO_) compared to the 184 disease subjects who lacked this allele (37.8±1.7% vs. 42.8±1.4%, p = 0.036).

**Conclusions/Significance:**

DRB1*1501 is more prevalent among IPF patients than normal subjects, and may be associated with greater impairment of gas exchange. These data are novel evidence that immunogenetic processes can play a role in the susceptibility to and/or manifestations of IPF. Findings here of a disease association at the HLA-DR locus have broad pathogenic implications, illustrate a specific chromosomal area for incremental, targeted genomic study, and may identify a distinct clinical phenotype among patients with this enigmatic, morbid lung disease.

## Introduction

Idiopathic pulmonary fibrosis (IPF) is a chronic, morbid, fibroproliferative lung disease that manifests with progressive pulmonary restriction and gas exchange abnormalities [1]. The age- and gender-adjusted prevalence of this disease in the U.S. has been recently estimated as 28 to 63 cases per 100,000 persons, and may be increasing [2]. IPF has a dismal prognosis, with a median survival of approximately 3 years after diagnosis, and no medical treatments have proven efficacy [1], [3].

Despite extensive investigation, the etiology of IPF remains obscure. Although the pulmonary fibrosis that characterizes this disease is often considered to be uniquely dissociated from inflammatory processes [3], studies of patient-derived specimens show that abnormal adaptive immune responses are common in IPF [4]–[18].

HLA allele frequencies are often aberrantly distributed among patients with immunologic disorders [19]–[24]. However, HLA characterizations of IPF populations have not been extensively pursued, particularly among disease cohorts that have been delineated by contemporaneous diagnostic criteria [1] and use of definitive, molecular allele typing.

We hypothesized that HLA frequency perturbations may also occur in IPF. Given the apparent prominence of CD4 T-cell responses in IPF [4]–[9], [14]–[17], we were singularly interested in evaluating the HLA Class II allele frequencies of these patients, since CD4 lymphocytes are predominantly HLA Class II-dependent [25]. Findings of HLA allele biases in IPF could have significant implications for the role of immunogenetic mechanisms in the pathogenesis of this disease.

## Methods

### Subjects

The initial (discovery) IPF cohort consisted of 79 consecutive patients with end-stage pulmonary disease who had molecular HLA allele determinations during evaluations for lung transplantations at the University of Pittsburgh (U. Pgh.), beginning in March 2006.

The subsequent disease validation cohorts were composed of IPF subjects for whom molecular HLA typing results and/or tissue specimens for HLA typing were available from the National Institute of Health (NIH) (n = 35), University of Chicago Medical Center (n = 32), Inova Fairfax Hospital (n = 20), and Stanford University Medical Center (n = 14). Subjects from Inova and Stanford were recipients of lung transplantations for end-stage IPF at their respective medical centers. IPF subjects from the NIH and University of Chicago were ambulatory clinic patients. In addition, data were compiled for 19 subjects who had lung transplantations for end-stage IPF at the U. Pgh. subsequent to the initial (discovery) analysis (now current thru October 17, 2010), and 76 ambulatory U. Pgh. IPF Clinic patients.

Diagnoses were prospectively established in the IPF subjects by expert, specialized pulmonary clinicians, blinded to these experimental studies, who analyzed all clinical information, including medical histories and physical exams, pulmonary function tests (PFTs), laboratory studies that included serologic tests for conventional autoimmune syndromes, rheumatologist evaluations, chest radiographs, and computerized tomography (CT) scans that were interpreted by radiologists blinded to other study results. All IPF study subjects fulfilled consensus diagnostic criteria [1]. None had clinical evidence or a past history of connective tissue diseases, drug toxicities, or occupational/environmental exposures associated with interstitial lung disease. Extensive histological evaluations of the diseased pulmonary explants were performed in all cases by blinded, expert lung pathologists. Subjects were excluded if they had histological patterns in their explanted lungs other than usual interstitial pneumonia (UIP) or end-stage fibrotic lung disease [26].

Right-heart catheterizations were routinely performed during assessments for lung transplantations (i.e., among U. Pgh., Inova, and Stanford IPF subjects) by cardiologists who were blinded to these HLA characterizations or knowledge of this study.

Controls consisted of normal Caucasian subjects who had prior molecular HLA typing in the course of other investigations at the U. Pgh. (n = 196) [27] and NIH (n = 41) [28], as well as an additional, prospectively recruited and analyzed U. Pgh. cohort (n = 48). All control subjects were healthy, ambulatory volunteers recruited for study by advertisement and/or solicitation.

Analyses are restricted to Caucasian subjects because <5% of lung transplantation recipients in the discovery cohort were members of minority groups, and HLA allele frequencies can vary greatly among racial/ethnic subpopulations [29], [30].

Subjects gave written informed consent for these studies that were approved by the respective Institutional Review Boards of all the participating medical centers (e.g., U. Pgh., NIH, U. Chicago, Inova, and Stanford).

### HLA Typing

HLA characterizations of the initial U. Pgh. IPF and normal cohorts were performed using DNA isolated from leukocytes, in sequence specific oligonucleotide probe assays [27]. HLA alleles among the NIH subjects (both IPF and normal) and the prospective U. Pgh. normal cohort (n = 48) were evaluated by polymerase chain reaction using sequence specific primers (PCR-SSP) (Invitrogen). The presence or absence of DRB1*15 was determined by PCR-SSP among validation IPF subjects at Chicago, Inova, Stanford, and the ambulatory U. Pgh IPF patients. HLA-DRB1* loci typing among those replication cohort specimens that were positive for DRB1*15, as well as high resolution typing of HLA-DRB1*15 per se were similarly performed by PCR-SSP. Previous study of common specimens confirmed complete concordance of PCR-SSP and oligonucleotide probe assays (n = 40).

### Statistical Methods

Allele-disease associations, as well as comparisons of other dichotomous variables, were established by Chi square. Logistic regression analyses were used to generate odds ratios and 95% confidence intervals. Two-group comparisons of continuous variables were made by two-sample t-test. Factorial ANOVA was used for three or more group comparisons of demographic and physiologic data, with post-hoc comparisons by Bonferroni/Dunn. Analyses were conducted with StatView v5.0.1 (SAS Institute, Cary, NC). Alpha (p) values <0.05 were considered significant. Data are depicted as means±SE.

## Results

### IPF Subjects

A total of 275 IPF subjects were studied here (Table 1). The IPF lung transplantation recipients from the U. Pgh., Inova, and Stanford had comparable pulmonary function abnormalities, although U. Pgh. and Chicago subjects tended to be older (Table 1). The extent of pulmonary restriction, ascertained by forced vital capacities as a percentage of predicted values (FVC%p), was less severe among ambulatory IPF subjects, compared to the transplant recipients (66±2% vs. 53±1%, respectively, p<0.0001). Similarly, the percentages of predicted values for single-breath carbon monoxide diffusing capacities (DL_CO_%p), an indicator of intrapulmonary gas exchange, were significantly greater in the ambulatory IPF patients (48±2%) than among those patients who had lung transplantations (33±1%) (p<0.0001).

**Table 1:** IPF Subject Characteristics.

	U. Pgh. Tx.	NIH	U. Chicago	Inova	Stanford	U. Pgh. Amb	Aggregate
n	98	35	32	20	14	76	275
Age (years)*	67±1	63±1	69±1	58±1	59±2	70±1	66±1
Males (%)	73	69	90	65	57	74	73
Smoking hx (%)	66	69	78	55	57	61	65
Status	Tx	Amb	Amb	Tx	Tx	Amb	Both
FVC%p**	52±2	74.0±4	65±2	55±4	55±5	63±2	60±1
DL_CO_%p***	32±1	54±3	43±3	36±4	35±4	48±2	41±1
DRB1*15^+^ (%)	36 (37)	12 (34)	10 (31)	9 (45)	4 (29)	24 (32)	95 (35)
DRB1*1501^+^ (%)	35 (36)	11 (31)	10 (31)	8 (40)	3 (21)	24 (32)	91 (33)

Tx: transplant recipients; Amb: ambulatory clinic outpatients; hx: history. U.Pgh. Tx includes the 79 subjects of the initial discovery cohort, as well as an additional 19 recipients of lung transplantations for end-stage IPF that have occurred at that institution since the initial discovery compilation. FVC%p denotes forced vital capacity, as a percentage of predicted normal values; DL_CO_%p denotes diffusing capacity for carbon monoxide as a percentage of predicted normal values. Values among U. Pgh. Tx, Inova, and Stanford subjects are based on last determinations immediately prior to their lung transplantations.

*<0.003 for U. Pgh. (both Tx. and OP) and/or U. Chicago vs. all others;

**<0.003 for NIH vs. all other groups; U. Pgh. Tx vs. all Amb subpopulations.

***p<0.004 for NIH vs. all Tx cohorts, U. Pgh. Tx vs. all Amb groups.

### HLA-DRB1*15 Prevalences

The initial compilation of HLA alleles for the discovery cohort of 79 U. Pgh. transplantation recipients showed the prevalence of DRB1*15 (calculated as the proportion of subjects who have either one or two copies of this allele) was greater among the IPF patients than in a normal control population [27] (Table 2). None of the other HLA Class II polymorphisms appeared to be significantly over-represented in these IPF patients (Table 2).

**Table 2:** HLA Class II Allele Prevalence in the Initial IPF Cohort.

DQB1* Alleles	IPF Prevalence	Control Prevalence	p value
02	29.1	40.6	0.07
03	54.4	55.7	0.70
04	3.8	7.8	0.23
05	29.1	31.8	0.67
06	51.9	40.1	0.08

DRB1* Alleles			
01	17.7	21.4	0.49
03	20.3	21.4	0.83
04	29.1	28.1	0.86
07	21.5	26.5	0.39
08	3.8	8.7	0.16
09	1.3	1.0	0.86
10	2.5	1.0	0.34
11	16.5	20.9	0.40
12	2.5	3.1	0.81
13	24.1	25.5	0.80
14	5.1	3.6	0.57
15	36.7*	23.0	0.02
16	3.8	5.1	0.64

HLA allele prevalences (the percentages of subjects with one or more copies of the allele) in the initial U. Pgh. IPF transplant recipient population (n = 79) were compared to those of a normal reference population (n = 196). DRB1*15 was the most over-represented of the common HLA Class II alleles among the IPF (bold) relative to the controls. These initial findings prompted further study by recruitments of IPF validation cohorts from four other medical centers and high resolution typing of the DRB1*15 allele (see text).

Based on these initial findings, validation cohorts were prospectively compiled, consisting of HLA data from IPF subjects at the NIH and results of DRB1*15 determinations using blood or tissue specimens from IPF patients at U. Chicago, Inova, Stanford, the IPF outpatient clinic at U. Pgh., as well as an additional volunteer U. Pgh. healthy control cohort. Those IPF and control specimens positive for DRB1*15 were further analyzed to characterize the specific alleles at this locus.

DRB1*15 was present in 64 normal subjects (23%), and in 95 IPF patients (35%) (p = 0.0015). The DRB1*1501 allele accounts for most DRB1*15 expression in normal Caucasians [29], and similar findings were present in the subjects here. DRB1*1501 accounted for all but 7 of the DRB1*15 occurrences among the controls and 4 of the IPF patients, and these exceptions were instead attributable to DRB1*1502.

**Figure 1:**
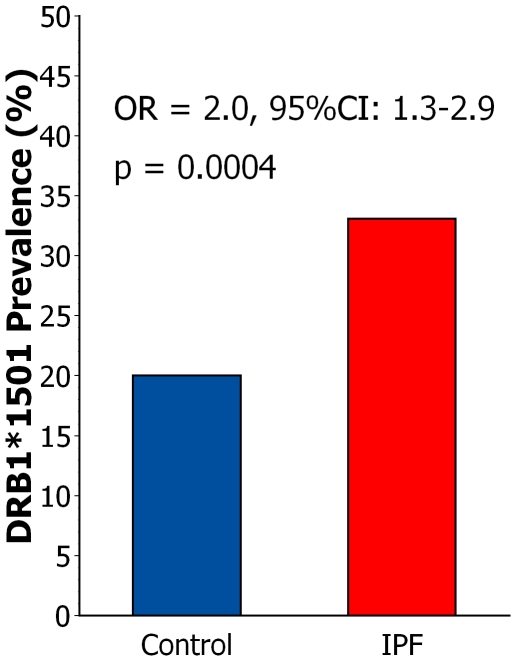
DRB1*1501 prevalence in IPF and controls. DRB1*1501 was significantly over-represented in the cumulative IPF population (n = 275) compared to healthy controls (n = 285).

The prevalence of DRB1*1501 within the aggregate IPF replication cohort (32.1%) was comparable to that of the original, discovery subjects (35.4%) (p = 0.60). Likewise, there was no significant difference of DRB1*1501 prevalence between IPF ambulatory patients (31.5%) and IPF transplant recipients (34.8%) (p = 0.55). The relative distributions of DRB1*1501 genotype frequencies among the respective control and IPF subpopulations were also similar in both discovery and replication cohorts (Table 3). However, the overall prevalence of this allele among IPF patients was significantly greater than that of the normal controls (Figure 1).

**Table 3:** Genotype distribution of DRB1*1501.

Cohort	Discovery		Replication	
Subjects	Control (n = 196)	IPF (n = 79)	Control (n = 89)	IPF (n = 196)
Genotype:				
DRB1*1501/DRB1*1501	1 (0.5%)	3 (3.8%)	1 (1.1%)	3 (1.5%)
DRB1*1501/other	38 (19.4%)	25 (31.6%)	17 (19.1%)	60 (30.6%)
other/other	157 (80.1%)	51 (64.6%)	71 (79.8%)	133 (67.9%)
p value*	0.0079		0.12	
Allele prevalence (any DRB1*1501 present) p value	0.003		0.035	

Parentheses denote percentages within respective control and IPF subject cohorts. *denotes p values for the 3×2 chi-square comparisons of the three genotype distributions among controls vs. IPF within the discovery or replication cohorts, respectively. The p value of the cumulative 3×2 chi-square comparison (the three genotype group distributions among all controls vs. all IPF subjects) is 0.0015.

Five IPF patients were known to have first-degree relatives who died with lung disease(s) that could possibly have been IPF. Two of these patients had DRB1*1501. Inasmuch as was known, the remaining IPF cases were due to sporadic disease [1].

Four (4) pulmonary transplantation recipients with pre-operative diagnoses of IPF were excluded from analyses on the basis of lung explant histological evaluation that instead showed single cases each of: pulmonary vasculitic changes suggestive of a clinically unsuspected autoimmune disorder; predominant lymphangitic non-necrotizing granulomas and lymphocytic infiltrates more consistent with hypersensitivity pneumonitis or sarcoidosis; extensive necrotizing granulmatous bronchitis that was highly suggestive of an infectious etiology; and a clinically unsuspected, histoplasma pneumonitis. One of these excluded subjects (25%) had DRB1*1501.

### DRB1*1501 Associations with Clinical Parameters

**Figure 2:**
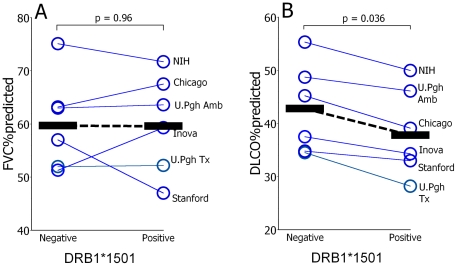
Associations of DRB1*1501 and lung function. A.) Forced vital capacities, as percentages of predicted normal values (FVC%predicted), did not show a consistent association with the presence or absence of DRB1*1501 among the IPF subjects. Aggregate mean values are denoted by horizontal bars linked by dashed lines. B.) Diffusing capacities, as percentages of predicted normal values (DL_CO_%predicted), were decreased among those IPF subjects with DRB1*1501 vs. those patients who did not have this allele, at each participating center. These differences were significant in comparisons of the cumulative (aggregate) IPF populations, despite considerable overall differences of DL_CO_%predicted values (e.g., “noise”) between the various participating medical centers (see also Table 1).

There were no apparent associations between the presence (n = 91) or absence (n = 184) of DRB1*1501 among the IPF patients and either age (66±1 vs. 67±1 years) or gender (75% vs. 73% males), for the DRB1*1501^+^ and DRB1*1501^null^ subjects, respectively. Similarly, FVC%p did not consistently differ between the IPF patients with and without DRB1*1501 (Figure 2A).

However, DL_CO_%p values were consistently decreased among the DRB1*1501^+^ IPF subjects at each study site (Figure 2B). The mean comparative decrement of this clinically relevant physiologic parameter (31,32) was ∼12% among the DRB1*1501^+^, compared to IPF subjects who did not have this allele. This intergroup difference seemed unlikely to be a cryptic result of smoking per se, as near equal proportions of IPF subjects with and without DRB1*1501 had smoking histories (67% and 64%, respectively) and cumulative smoke exposures (23±3 and 21±2 pack-years, respectively). DL_CO_ abnormalities can also be attributable to secondary effects of pulmonary artery hemodynamics [33]. Nonetheless, we found no intergroup differences of pulmonary artery pressures based on the presence or absence of DRB1*1501 among those IPF patients who had right heart catheterizations (i.e., the transplantation recipients from the U. Pgh., Inova, and Stanford), with respect to systolic (41±2 vs. 43±2 mm Hg, respectively), diastolic (14±1 vs. 15±1 mm Hg, respectively) or mean pressures (25±1 vs. 26±1 mm Hg, respectively).

## Discussion

These findings show the HLA Class II allele DRB1*1501 is over-represented among Caucasian IPF subjects with highly variable disease severities at multiple U.S. medical centers (Figure 1). The presence of DRB1*1501 in IPF patients also seems associated with greater magnitudes of gas exchange impairment (Figure 2B). The present findings implicate immunoregulatory elements within the HLA Class II complex in the pathogenesis of IPF. These data are also consistent with the hypothesis that the development of IPF likely involves interactions between environmental agent(s) and genetic factors [34], [35], a disease paradigm also common to many other disorders, notably including those caused by aberrant immune processes [19]–[24].

Two distinct mechanisms may account for the frequently observed associations between unique HLA polymorphisms and various disease syndromes [19]–[24]. First, HLA molecules are requisite effectors for presentations of peptide antigens to the T-cells that initiate adaptive immune responses, but each distinct HLA allele has a restricted peptide binding motif [25]. Hence, HLA haplotype inheritance determines the finite repertoire of antigens that can evoke T-cell responses in an individual. Although critical for host defense, these adaptive immune responses may be deleterious if, as an example, the antigen is a self-protein (autoantigen), or one that evokes a cross-response to a self-protein [25], [36], [37]. In contrast, individuals lacking these specific, “permissive” HLA alleles do not present those particular antigens, and do not initiate the deleterious response(s).

Alternatively, over-representation of a specific HLA molecule(s) in a disease cohort may be essentially unrelated to the unique antigen presentations of that HLA per se, but is instead a genetic “marker” denoting the presence of a pathogenic immunomodulatory gene(s) that is(are) in strong linkage disequilibrium (LD) with that HLA allele [21], [38].

The human major histocompatibility (MHC) complex on chromosome 6p21.31 is characterized by the presence of numerous, extraordinarily polymorphic HLA alleles, and many other proximate immunoregulatory genes that are often in very strong LD [19]–[21], [26], [38]. DRB1*1501 is in nearly complete LD with DQA1*0102 and DQB1*0602 among Caucasians, and is the single most frequent HLA Class II haplotype in this racial group [29]. Over-representation of the DRB1*1501 HLA allele (and/or the DRB1*1501-DQA1*0102-DQB1*0602 haplotype) is also one of the most frequently reported genetic findings of patients with diverse immunologic diseases, including Goodpasture's syndrome, multiple sclerosis, systemic lupus erythematosus (SLE), and sarcoidosis [19]–[24]. Because of the very strong LD within this haplotype, it is difficult to precisely identify the particular disease-associated HLA allele among them, or distinguish the contributions of these HLA from other interspersed immunoregulatory elements, in lieu of focused, high-resolution genomic studies [21], [38], [39].

Almost all previous HLA characterizations of IPF patients date from before the development of precise and definitive molecular methodologies that distinguish these alleles [40]–[45] (Table 4). Moreover, only two of these early serologic-based determinations examined even a very restricted repertoire of the many, since-discovered, Class II alleles [44], [45]. Furthermore, diagnostic criteria for IPF have evolved considerably during the intervening years [1], raising potential concerns about the case definitions of the earlier study populations. The numbers of subjects among those investigations were also usually quite small (Table 4), severely limiting their power to detect intergroup differences. Despite these potential limitations, however, several of those earlier investigations indicated HLA allele frequency perturbations may be present in IPF [40], [43]–[45], although this finding was not invariable [41], [42]. In particular, one of these earlier studies indicated that DR2, a serologic correlate of HLA-DRB1*15 and, generally much less frequently, HLA-DRB1*16 gene products, appeared to be over-represented in IPF subjects (45), a finding which may be congruent with the current results.

**Table 4:** Published studies of HLA allele frequencies in IPF.

Loci	#Alleles tested	Molecular diagnoses	IPF (n)	Abnormal in IPF?	year	ref
?	?	No	20*	Yes HLA12	1976	40
-A, -B	24	No	32	no	1977	41
-A, -B	35	No	33	no	1978	42
-A, -B, -C	36	No	50	Yes: B8	1978	43
-A, -B, -C, -Dw	32	No	38*	Yes: B15 and Dw6	1979	44
-A, -B, -C, -DR	65 (total in all loci)	No	20	Yes: DR2	1983	45
-A, -B, -DR, -DQ	45 multiple	Yes	75	Yes, multiple Class I and Class II alleles and haplotypes (but not DRB1*15)	2005	46

*included patients with other autoimmune syndromes. Approximately fifty (50) HLA-A, 85 HLA-B, 45 HLA-C, 44 HLA-DR, and 16 HLA-DQ distinct polymorphisms (alleles and suballeles) are currently known to be expressed in Caucasian populations [29].

To our knowledge, contemporary analogous analyses using molecular techniques and current IPF case definitions are limited to a single cohort study of Mexican patients that reported various HLA alleles, including DRB1*01, DRB1*04, and DRB1*14, were over-represented in IPF [46]. We did not see abnormal frequencies of those particular DRB1* alleles (DRB1*01, *04, or *14) in our IPF population (Table 2). Conversely, the frequencies of DRB1*15 alleles in both the IPF and normal control populations of that previous study [46] were several-fold less than that measured here, and were also much less than frequencies reported within other large Caucasian control populations [22], [24], [29], [30]. The seeming discrepancy between that previous report and the present findings may be attributable to the often considerable variability of HLA allele frequencies among different races and ethnicities [19]–[21], [29].

The present study has several unique aspects. To our knowledge, the number of subjects with this uncommon disease that were analyzed here is unprecedented among analogous investigations (Table 4). The findings of DRB1*15 over-representation in the discovery U. Pgh. cohort with end-stage lung disease was also uniquely validated in prospective replications of subjects from four other participating medical centers, and included IPF patients with highly variable disease severities. The present study is also unusual in that, in addition to adherence with current diagnostic criteria [1], extensive histological evaluations of entire lung explants were available for the many disease subjects here who had lung transplantations, thereby further ensuring accuracy of these case definitions.

Other findings here that DL_CO_ tended to be comparatively less among those IPF patients with DRB1*1501 (Figure 2B), particularly in the absence of confounding by intergroup differences of smoking exposures or pulmonary artery pressures, could perhaps imply that the role ultimately played by this immunogenetic factor(s) may have a singularly virulent effect with respect to the lung injury mechanisms that result in gas exchange impairment. Decrements of DL_CO_ are widely used indices of disease activity and prognosis [31], and may be useful ancillary indications for lung transplantation among IPF patients [32].

Although IPF is not widely considered to be an immunologic disorder, recent studies of patient-derived clinical specimens reveal the presence of abnormal adaptive immune responses among those afflicted by this disease. The majority of IPF patients have IgG autoantibodies against various autoantigens that are typically distinct from those described in classical autoimmune syndromes (e.g., SLE, scleroderma, etc.) [5]–[8], [9], [14], and the presence of particular autoantibody responses in individual IPF patients have been associated with clinical manifestations of their lung disease [4], [5], [9]. In addition, T-cells among IPF patients show increased extents of prior activation, enhanced production of various inflammatory and/or pro-fibrotic mediators (e.g., TGF-β1), and impaired regulatory (T_reg_) function [10]–[14], [17]. Moreover, first-degree relatives of patients with familial IPF show intrapulmonary infiltrations of activated CD4 T-cells many years prior to the development of clinically-evident lung abnormalities [15]. Activated, pro-inflammatory dendritic cells with augmented ability to present antigens to T-cells also accumulate in the pulmonary parenchyma of IPF patients [18]. CD4 T-cell oligoclonal proliferation is extensive in the lungs and periphery of IPF patients, a highly specific finding of repetitive lymphocyte stimulation by a restricted set of conventional peptide antigens [13], [14]. Abnormal proportions of phenotypically and functionally distinct CD4^+^CD28^null^ T-cells, the daughter progeny of repetitive antigen-driven lymphocyte proliferations, and a frequent and specific finding of chronic immunologic diseases [47], [48], are also present in the circulation (and lungs) of IPF patients singularly destined for poor outcomes [16]. Moreover, intrapulmonary peptide antigen(s) isolated from diseased IPF lungs uniquely drive proliferations of autologous CD4 T-cells [14].

Considerable efforts have been directed towards discovery of the genetic determinants for IPF [34], [35]. The present data show that the HLA Class II complex, a unique chromosomal region with extreme polymorphism, unusually strong, nonrandom LD, and a high density of diverse immunoregulatory genes [21], [38], [39], include one or more loci involved in IPF pathogenesis. These findings justify further, specific characterizations of targeted HLA region genes and polymorphisms among IPF cohorts, and correlative clinical and immunologic functional studies, in order to ultimately discern the processes that contribute to development and/or progression of this devastating, intractable disease.
